# Preliminary findings on the effect of childhood trauma on the functional connectivity of the anterior cingulate cortex subregions in major depressive disorder

**DOI:** 10.3389/fpsyt.2023.1159175

**Published:** 2023-04-17

**Authors:** Bei Rong, Guoqing Gao, Limin Sun, Mingzhe Zhou, Haomian Zhao, Junhua Huang, Hanling Wang, Ling Xiao, Gaohua Wang

**Affiliations:** ^1^Department of Psychiatry, Renmin Hospital of Wuhan University, Wuhan, Hubei, China; ^2^Institute of Neuropsychiatry, Renmin Hospital of Wuhan University, Wuhan, Hubei, China; ^3^Xi’an Jiaotong-Liverpool University, Suzhou, Jiangsu, China; ^4^Taikang Center for Life and Medical Sciences, Wuhan University, Wuhan, Hubei, China

**Keywords:** major depressive disorder, childhood trauma, resting state, functional connectivity, anterior cingulate cortex

## Abstract

**Objectives:**

Childhood trauma (CT) is a known risk factor for major depressive disorder (MDD), but the mechanisms linking CT and MDD remain unknown. The purpose of this study was to examine the influence of CT and depression diagnosis on the subregions of the anterior cingulate cortex (ACC) in MDD patients.

**Methods:**

The functional connectivity (FC) of ACC subregions was evaluated in 60 first-episode, drug-naïve MDD patients (40 with moderate-to-severe and 20 with no or low CT), and 78 healthy controls (HC) (19 with moderate-to-severe and 59 with no or low CT). The correlations between the anomalous FC of ACC subregions and the severity of depressive symptoms and CT were investigated.

**Results:**

Individuals with moderate-to severe CT exhibited increased FC between the caudal ACC and the middle frontal gyrus (MFG) than individuals with no or low CT, regardless of MDD diagnosis. MDD patients showed lower FC between the dorsal ACC and the superior frontal gyrus (SFG) and MFG. They also showed lower FC between the subgenual/perigenual ACC and the middle temporal gyrus (MTG) and angular gyrus (ANG) than the HCs, regardless of CT severity. The FC between the left caudal ACC and the left MFG mediated the correlation between the Childhood Trauma Questionnaire (CTQ) total score and HAMD-cognitive factor score in MDD patients.

**Conclusion:**

Functional changes of caudal ACC mediated the correlation between CT and MDD. These findings contribute to our understanding of the neuroimaging mechanisms of CT in MDD.

## Introduction

Major depressive disorder (MDD) is a prevalent psychiatric disease affecting approximately 15–18% of the general population around the world (1). Globally, depression was the leading contributor to disability in 2017 according to the World Health Organization, and its prevalence continues to increase (2). To date, the neurobiological mechanisms underlying depression remain incompletely characterized. Consequently, it is imperative to understand the neurobiological mechanisms of MDD and identify effective therapies for depression.

Neuroimaging studies point to the anterior cingulate cortex (ACC), a region involved in emotions, information processing, and regulation, which serves a vital role in the pathophysiology of depression (3, 4). Previously published studies ubiquitously found that abnormalities of gray matter volume (5–11), white matter volume (12, 13), and functional activity (14–17) of the ACC could be responsible for depression in MDD patients. In addition, accumulating evidence from previous studies indicated that the alterations in the structural and functional activity of ACC have emerged as a promising predictor of the effectiveness of depression treatment. For instance, the increased volume ratio of ACC was correlated to the improvement of depressive symptoms after ECT (18). After Cognitive Behavioral Therapy (CBT), the reduction in depressive symptoms was positively related to an increase in ACC volume (19). Additionally, changes in baseline metabolic activity in the ACC can also predict the effectiveness of antidepressant treatment (20). Evidence suggests that deep brain stimulation treatment targeted at white matter tracts adjacent to the subgenual ACC can relieve depression symptoms (21, 22). Thus, the structural and functional activity of ACC is crucial to the diagnosis, prognosis, and therapy of MDD.

There is wide recognition that childhood trauma (CT) contributes to MDD risk. Patients with depression who suffer maltreatment are more likely to develop chronic diseases and have worse treatment outcomes (23). In previous studies, the volume of ACC has been shown to be reduced in people with CT (24–28). These changes in the volume of ACC in CT are consistent with the findings reported in MDD studies (29), indicating that CT and MDD may be linked by the ACC. In spite of this, it remains unclear how CT affects MDD physiologically. Recently, there has been an increased focus on brain connectivity and how it relates to psychopathology as well as changes in gray matter structure and function (30, 31). By using functional magnetic resonance imaging (fMRI), we can detect the temporal organization of the functional brain circuits on a large scale organization of neural functional brain circuits by detecting temporally correlated, spontaneous variations in blood oxygen levels; this is also termed resting-state functional connectivity (rsFC) (32).

Previous evidence based on FC has suggested that MDD patients have extensively aberrant FC between the ACC and multiple areas of the brain (9, 14, 17). The structure and function of the ACC, however, are considered to be heterogeneous and can generally be divided into five subregions associated with distinct functions (33–35). Recent studies have investigated changes in ACC subregions in MDD (36–40). A previous study showed that the subregions of ACC (including subgenual, perigenual, and caudal ACC) exhibited a reduction in FC with the key hubs in the default mode network (DMN), and a decreased FC of the caudal ACC to the precuneus has a negative link to depression symptoms (37). Additionally, a reduced FC of subgenual ACC to the middle frontal gyri (MFG) and inferior frontal gyri (IFG) has been related to rumination in medication-naïve, first-episode MDD adolescents (36). One study demonstrated that the FC of perigenual ACC-superior frontal gyrus (SFG) mediates the association between anhedonia and sleep quality in MDD patients (41). As a result, MDD may be characterized by the alteration of functional integration in subregions of the ACC. As with depression research, reduced subregions of ACC FC have been observed in individuals with CT, both clinically and non-clinically. Research on 64 late adolescents found that a higher level of CT was correlated to a lower FC of the subgenual ACC to the amygdala (42). However, a study of healthy adolescents found that adolescents with CT exhibited the reduced FC between the subgenual ACC and the fronto-parietal network (FPN) (including the dorsolateral prefrontal cortex (DLPFC), supramarginal gyrus (SMG), and cuneus) (43). In other studies, CT has been shown to be negatively associated with the FC of the rostral ACC to the amygdala and hippocampus (44, 45). These studies suggest that the alterations in the FC of ACC subregions were also related to the history of CT. To our knowledge, no studies have examined the alteration in the FC of ACC subregions in MDD patients with moderate-to-severe CT and with no or low CT.

This study aimed to investigate whether and how CT, current depression, and both affect FC patterns of ACC subregions in the first-episode, drug-naïve MDD patients compared with healthy controls (HCs). In addition, we also examined the association between the aberration in FC of ACC subregions and the severity of depressive symptoms. Finally, we explored the possible effect of ACC subregions FC in the associations between CT and depressive symptoms using a mediation analysis model.

## Materials and methods

### Participants

Sixty first-episode, drug-naive MDD patients and 78 gender- and age-matched HC were included in the present study. All individuals with MDD were recruited from the department of psychiatry, Renmin Hospital of Wuhan University from April 2021 to July 2022. The recruitment of matched healthy control subjects was accomplished through advertisements. Patients were diagnosed using the Structured Clinical Interview for the Diagnostic and Statistical Manual of Mental Disorders (DSM-IV) (SCID). The 17-item Hamilton Depression Scale (HAMD-17) and 14-item Hamilton Anxiety Scale (HAMA) were used to measure the depression and anxiety levels of all participants. The inclusion criteria for MDD patients included: (1) meeting the DSM-IV diagnostic criteria for MDD, (2) having first-episode MDD with no history of treatment, including psychoactive drugs, psychotherapy, and so on, (3) being between the age of 18 and 65 years, (4) being right-handed, and (5) having a total score of HAMD-17 > 17. HCs were admitted to this study if they met the following criteria: (1) were without any major psychiatric illness and had no family history of major psychiatric illnesses, (2) were between the age of 18 and 65 years, and (3) were right-handed. Any participant who met any of the following criteria was excluded: (1) they were under the age of 18 or over 65 years old, (2) had a history of another DSM-IV Axis I psychiatric disorder, (3) had any neurological disorders or head injury, (4) had a substance abuse or dependency history, or (5) had other contraindications for imaging scanning. The five subscales of the HAMD-17 were scored as anxiety/somatization, weight, cognitive disturbance, retardation, and sleep disruption (46). The Chinese version of the Childhood Trauma Questionnaire (CTQ) was utilized to assess CT (47). It is a 28-item self-reported questionnaire that evaluates five aspects of childhood trauma, including emotional neglect (EN), physical neglect (PN), emotional abuse (EA), physical abuse (PA), and sexual abuse (SA). The cut-off scores of each subscale for moderate-to-severe trauma are: PN ≥ 10, PA ≥ 10, EN ≥ 15, EA ≥ 13, and SA ≥ 8. In this study, individuals scoring above the subscale threshold (moderate-to-severe) were considered to have been exposed to corresponding CT (48). All participants provided informed consent for this study and were recruited. This current study received approval from the Ethics Committee of Renmin Hospital of Wuhan University.

### MRI acquisition

All participants’ imaging data were acquired on a 3.0T GE Signa HDx MRI scanner at Renmin Hospital of Wuhan University. In order to maintain motionlessness, participants were informed to keep their eyes closed and keep thinking of nothing special. High-resolution T1-weighted structural images were obtained by gradient-echo sequence: repetition time (TR) = 8.5 ms; echo time (TE) = 3.2 ms; flip angle = 12°; slice thickness = 1.0 mm; gap = 0.0 mm; field of view (FOV) = 256 mm × 256 mm; matrix = 256 × 256; voxel size = 1.0 mm × 1.0 mm × 1.0 mm; 176 slices. An echo planar imaging (EPI) sequence was used to acquire resting-state functional MRI data: TR = 2,000; TE = 30 ms; flip angle = 90°; voxel size = 3.4 mm × 3.4 mm × 4.0 mm; slice thickness = 4.0 mm; gap = 0 mm; FOV = 220 mm × 220 mm; matrix = 64 × 64; 36 slices; resulting in a total of 240 volumes acquired.

### MRI data preprocessing

Preprocessing was carried out with Statistical Parametric Mapping (SPM12)^1^ and Data Processing Assistant for Resting-State fMRI (DPARSF V5.2) (49). To ensure magnetization stability, the first 10 volumes of each participant were removed. Slice timing correction was conducted to account for interleaved acquisition. Functional images were realigned to correct the head motion. A participant with a maximum displacement over 3 mm or rotation over 3° was excluded from the analysis. Subject-wise 3D T1-weighted structural images were co-registered with the mean functional images. Subsequently, each participant’s functional images were normalized to the standard Montreal Neurological Institute (MNI) space by using the transformation co-registered T1 to MNI space and resampled to 3.0 mm × 3.0 mm × 3.0 mm voxels. Spatial smoothing was performed using a 6 mm full-width half-maximum (FWHM) isotropic Gaussian kernel. Linear detrending was applied to remove low-frequency drift. Several confounding factors including WM signal, CSF signal, and Friston-24 head motion parameters were regressed to allow for minimal effects of possible motion-related confounds. A temporal bandpass filter at 0.01–0.1 Hz was applied to control physiological high-frequency noise. There were no subjects excluded for excessive head motion >3 mm displacement or >3°.

### Functional connectivity measurement

**FIGURE 1:**
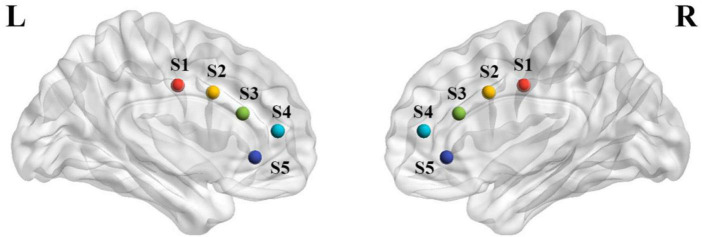
The map of five ACC subregions in each hemisphere. Five seeds for left and right hemisphere to represent different ACC functional subdivisions, including caudal ACC (L/R-Sl, blue, MNI = ±5, −10, 37), dorsal ACC (L/R-S2, peacock blue, MNI = ±5, 10, 33), rostral ACC (L/R-S3, green, MNI = ±5, 27, 21), perigenual ACC (L/R-S4, orange, MNI = ±5, 47, 11), and subgenual ACC (L/R-S5, red, MNI = ±5, 34, −4). All these seeds were defined in the Montreal Neurological Institute (MNI) coordinates with the spheres of 4 mm.

Using the DPABI toolbox, images were processed and then imported for seed-to-voxel FC analysis. In the present study, a total of 10 spherical regions of interest (ROIs) of 5 mm radius were identified for bilateral ACC, and bilateral caudal ACC (±5, −10, 37), dorsal ACC (±5, 10, 33), rostral ACC (±5, 27, 21), perigenual ACC (±5, 47, 11), and subgenual ACC (±5, 34, −4) were created to examine the seed-based, whole-brain, voxel-wise functional connectivity patterns of each subregion of the ACC (34, 35). The map of the 10 ROIs is displayed in Figure 1. For each subject, the time series of all voxels within each ROI was extracted and subsequently averaged to calculate Pearson’s correlation with the time series of all other voxels in the whole brain. Then Fisher’s r-to-z transformation was utilized to ensure the normality of FC. Thus, each FC map of ROIs was obtained. Then two-sample *t*-test was utilized to compare the differences in the FC map of ROIs between the MDD and HC groups after controlling for confounders of gender, age, education, and head motion. The threshold *p* < 0.001 at the voxel level and *p* < 0.05 at the cluster level (two-tailed), with Gaussian random field (GRF) theory, was taken to indicate the statistical significance.

### Statistical analysis

The Shapiro-Wilkes normality test and Levene’s test were applied to evaluate variance distribution and homogeneity, respectively. Independent samples *t*-test or Mann-Whitney U test (for non-Gaussian distributions) were used to compare the demographic and behavioral variables between the two groups. One-way ANOVA test with Bonferroni-test or Welch-ANOVA with Games–Howell post-test (the variances were not equal) was used to evaluate demographic and behavioral variables comparisons among the four groups. The Chi-square test was applied to assess the gender differences. All the numerical data are expressed as the mean [standard deviation (SD)]. The significance level was set at *P* < 0.05 (SPSS 23.0; SPSS Inc., Chicago, IL, USA).

To separately test the main effect of diagnosis and CT level and the interaction between diagnosis and CT level on depression and anxiety level, 2 (diagnosis: MDD and HC) × 2 (CT level: moderate-to-severe and no or low) two-way ANCOVAs were conducted, controlling for mean framewise displacement (FD). Demographic differences among the four groups were not significant.

Similarly, significant differences in the seed-to-voxel FC maps created by each seed among the four groups were investigated using 2 (diagnosis: MDD and HC) × 2 (CT level: moderate-to-severe, no or low) ANCOVAs, and gender, age, education level, and mean FD were included as covariates. Significant clusters for all maps comparisons were identified with a Gaussian random field (GRF) theory with a cluster level at *P* < 0.05 and a voxel-level threshold of *P* < 0.001 for multiple comparisons.

Partial correlations analysis was used to assess whether FCs were the main effect of CT level and were correlated with depressive levels, CTQ total score, and subscales score after controlling for age, gender, years of education, mean FD, and duration of illness (only in the MDD group). Multiple comparisons were performed using a False discovery rate (FDR) < 0.05. Then, mediation analysis was utilized by using Haye’s bootstrapping method (PROCESS macro based on SPSS model 4, utilizing 5000 bootstrap samples to estimate the 95% confidence interval) (50) to test the influence of FC alterations in the subregions of ACC on the relationship between CT and depressive symptoms in the MDD group, controlling for gender, age, education level, and mean FD.

## Results

### Demographic and clinical characteristics

The demographic and clinical characteristics of the four groups are summarized in Table 1. No significant differences were found in age, gender, mean FD, and years of education among the four groups after Bonferroni correction (*p* < 0.05/6 = 0.0083) in this study. The disease duration has no difference between the MDD_moderate-to-severe CT group and MDD_no or low CT group.

**TABLE 1:** Demographic and neuropsychiatric characteristics among four groups.

	MDD		HC			
	**Moderate-to-severe CT (*N* = 40)**	**No or low CT (*N* = 20)**	**Moderate-to-severe CT (*N* = 19)**	**No or low CT (*N* = 59)**	**F/t/χ^2^**	***P***
**Demographic characteristics**						
Age (years)	26.40 (5.38)	26.75 (8.81)	26.95 (7.83)	29.20 (8.94)	1.23^a^	0.302
Gender (Male/female)	32 (80%)	16 (80%)	11 (57.89%)	42 (71.19%)	3.80^a^	0.283
Education (years)	15.38 (1.73)	15.25 (2.97)	16.90 (2.18)	15.80 (2.30)	2.35^a^	0.076
Mean FD (mm)	0.06 (0.04)	0.06 (0.03)	0.06 ± 0.03	0.06 (0.03)	0.02^a^	0.997
Disease duration (month)	6.48 (4.12)	7.05 (4.27)	–	–	0.00	0.617
**Clinical characteristics**						
HAMD-17	26.55 (4.62)	24.90 (6.28)	1.68 (1.42)	0.90 (1.21)	706.55	<0.0011
HAMA	17.23 (2.87)	16.15 (2.52)	1.58 (2.12)	1.59 (1.78)	287.98	<0.0011
CTQ-EA	11.05 (4.38)	7.75 (2.17)	7.90 (3.07)	5.95 (1.41)	24.66	<0.0011
CTQ-PA	7.33 (2.77)	5.45 (0.76)	6.42 (3.11)	5.37 (0.61)	9.41	<0.00101
CTQ-SA	6.10 (2.57)	5.15 (0.67)	5.84 ± 2.85	5.15 (0.58)	3.26	0.032
CTQ-EN	16.38 (3.71)	9.85 (2.46)	11.05 (4.08)	8.14 (2.49)	66.35	<0.001
CTQ-PN	11.38 (3.59)	6.95 (1.85)	10.00 (2.67)	5.78 (1.25)	49.32	<0.001
CTQ-Total	52.26 (11.14)	35.15 (4.88)	41.21 ± 8.05	30.39 (4.18)	92.42	<0.001

Means with standard deviations in parentheses. F//t/χ^2^: Variables of age, years of education, mean FD, HAMD-17, HAMA-14, and CTQ assessments were tested by one-way ANOVA or Welch-ANOVA as indicated by F; ^a^analysis by one-way ANOVA; gender was calculated using chi-square test as indicated by χ^2^; disease duration was tested by two-sample *t*-test as indicated by t. Significant *post-hoc* tests (*p* < 0.05, Bonferroni corrected or Games–Howell corrected): HAMD-17: MDD_moderate-to-severe CT = MDD_no or low CT > HC_moderate-to-severe CT = HC_no or low CT; HAMA: MDD_moderate-to-severe CT = MDD_no or low CT > HC_moderate-to-severe CT = HC_no or low CT; CTQ_EA: MDD_moderate-to-severe CT > MDD_no or low CT = HC_moderate-to-severe CT > HC_no or low CT; CTQ_PA: MDD_moderate-to-severe CT = MDD_no or low CT = HC_moderate-to-severe CT = HC_no or low CT; CTQ_SA: MDD_moderate-to-severe CT > MDD_no or low CT = MDD_moderate-to-severe CT = HC_no or low CT; CTQ-EN: MDD_moderate-to-severe CT > MDD_no or low CT > HC_moderate-to-severe CT > HC_no or low CT; CTQ_PN: MDD_moderate-to-severe CT = HC_moderate-to-severe CT > MDD_no or low CT > HC_no or low CT; CTQ_Total: MDD_moderate-to-severe CT > HC_moderate-to-severe CT > MDD_no or low CT > HC_no or low CT. MDD, major depressive disorder; HC, healthy control; FD, frame displacement; HAMD-17, 17-items Hamilton Depression Scale; HAMA, Hamilton Anxiety Scale; CTQ-EA, Childhood Trauma Questionnaire-emotional abuse; CTQ-PA, Childhood Trauma Questionnaire-physical abuse; CTQ-SA, Childhood Trauma Questionnaire-sexual abuse; CTQ-EN, Childhood Trauma Questionnaire-emotional neglect; CTQ-PN, Childhood Trauma Questionnaire-physical neglect; CTQ-Total, Childhood Trauma Questionnaire total score.

### Diagnosis and CT level effects on clinical variables

There was a significant main effect of diagnosis on all depressive and anxiety levels, with MDD patients exhibiting severe depressive and anxiety symptoms compared to the HCs. The main effect of CT was identified on the HAMD-17 total score, and the HAMD-cognitive disturbance factor indicated that the subjects with moderate-to-severe CT displayed higher levels of depressive and cognitive disturbance symptoms than those with no or low CT. The significant main effect of diagnosis and the interaction effect of diagnosis × CT on HAMD total score was demonstrated only in the HC group; the individuals with moderate-to-severe CT exhibited higher levels of depressive symptoms than individuals with no or low CT. No significant main effect of CT or the interaction effect of diagnosis by CT on the HAMD-anxiety factor, HAMD-weight factor, HAMD-retardation factor, HAMD-sleep disorder factor, and HAMA were identified (Table 2).

**TABLE 2:** Diagnosis and childhood trauma effect on 17-items Hamilton Depression Scale (HAMD-17) and subscales.

Characteristics	MDD		HC		ANOVA								
	**Moderate-to-** **severe CT**	**No or low** **CT**	**Moderate-to-** **severe CT**	**No or low** **CT**	**Effect of diagnosis**			**Effect of CT**			**Effect of diagnosis × CT**		
	***N* = 40**	***N* = 20**	***N* = 19**	***N* = 59**	**F**	***P***	**Eta^2^**	**F**	***P***	**Eta^2^**	**F**	***P***	**Eta^2^**
HAMD-17	26.55 (4.62)	24.90 (4.50)	2.05 (1.22)	0.95 (1.20)	1237.50	<0.001	0.91	14.91	<0.001	0.10	5.54	0.020^a^	0.04
HAMD-anxiety	6.85 (1.79)	6.70 (1.42)	0.79 (0.71)	0.46 (0.68)	467.93	<0.001	0.78	3.45	0.065	0.03	2.63	0.108	0.02
HAMD-weight	2.70 (1.57)	2.35 (1.66)	0.0 (0.00)	0.0 (0.00)	174.46	<0.001	0.57	0.84	0.362	0.01	0.40	0.550	0.00
HAMD-cognitive	5.20 (1.88)	4.85 (1.95)	0.47 (0.51)	0.10 (0.31)	520.92	<0.001	0.80	7.94	0.006	0.06	2.75	0.100	0.02
HAMD-retardation	7.60 (1.91)	7.30 (1.53)	0.31 (0.67)	0.10 (0.36)	1322.17	<0.001	0.91	3.72	0.056	0.03	1.36	0.246	0.01
HAMD-sleep	4.20 (1.49)	3.70 (1.69)	0.42 (0.69)	0.65 (0.08)	278.05	<0.001	0.68	2.50	0.12	0.02	0.02	0.88	0.00
HAMA	17.23 (2.87)	16.15 (2.52)	0.37 (0.68)	1.59 (1.78)	608.48	<0.001	0.82	0.31	0.58	0.00	0.31	0.58	0.00

Means with standard deviations in parentheses. HAMD-17, 17-items Hamilton Depression Scale; HAMD-anxiety, Hamilton Depression Scale-anxiety/somatization factor, a sum of item 10, 11, 12, 15 and 17 in HAMD-17; HAMD-weight, Hamilton Depression Scale-loss of the weight factor, item 16 in HAMD-17; HAMD-cognitive, Hamilton Depression Scale-cognitive disturbance factor, a sum of item 2, 3 and 9 in HAMD-17; HAMD-retardation, Hamilton Depression Scale-retardation factor, a sum of item 1, 7, 8 and 14 in HAMD-17; HAMD-sleep, Hamilton Depression Scale-sleep disorder factor, a sum of item 4, 5, and 6 in HAMD-17; HAMA, Hamilton Anxiety Scale; CT, childhood trauma; MDD, major depressive disorder patients; HC, healthy controls. ^a^Further simple effects analysis suggested that only in the HC group, individuals with moderate-to-severe CT exhibited a higher level of depressive symptoms than individuals with no or low CT (HC_moderate-to-severe CT vs. HC_no or low CT: *p* < 0.001; MDD_moderate-to-severe CT vs. MDD_no or low CT: *p* = 0.311).

### Diagnosis and CT level effects on FC

**FIGURE 2:**
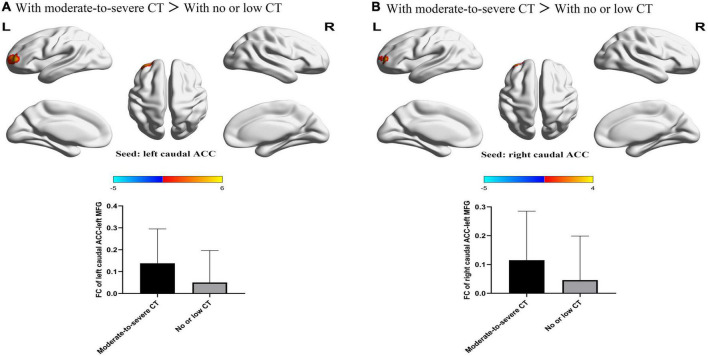
The significant main effect of CT level on FC between bilateral caudal ACC and the left MFG. **(A)** Individuals with a history of moderate-to-severe CT exhibited increased FC between the left caudal ACC seed and left MFG compared to individuals with no or low CT. **(B)** Individuals with a history of moderate-to-severe CT exhibited increased FC between the right caudal ACC and the left MFG compared to individuals with no or low CT. L, left; R, right; MDD, major depressive disorder; FC, functional connectivity; ACC, anterior cingulate cortex; MFG, middle frontal gyrus.

**FIGURE 3:**
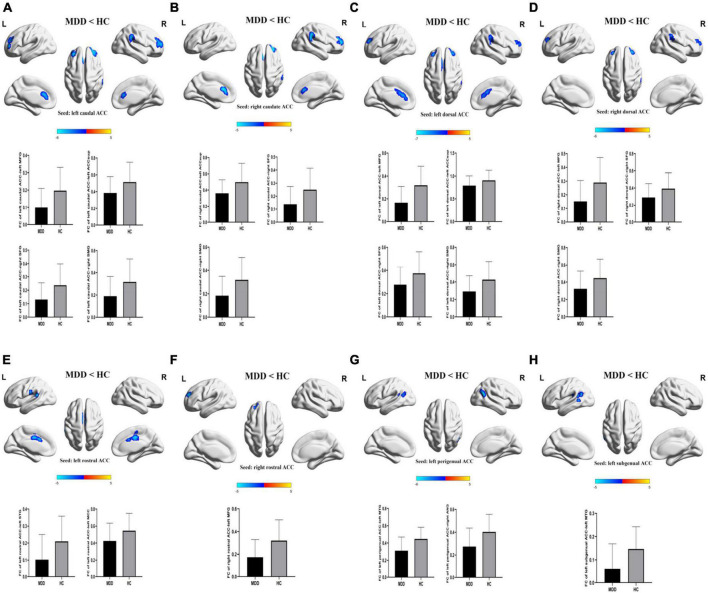
The significant main effect of diagnosis on FCs of ACC subregions. **(A–H)** MDD patients had decreased rsFCs of bilateral caudal ACC, bilateral dorsal ACC, bilateral rostral ACC, left perigenual ACC and left subgenual ACC compared to HC. Significance threshold was set at *p* < 0.001 at voxel level uncorrected, *p* < 0.05 at cluster level, with Gaussian random field correction (GRF). L, left; R, right; MDD, major depressive disorder; HC, healthy controls; FC, functional connectivity; CT, childhood trauma; ACC, anterior cingulate cortex; MFG, middle frontal gyrus; SFG, superior frontal gyrus; SMG, supra marginal gyrus; STG, superior temporal gyrus; MTG, middle temporal gyrus; ANG, angular gyrus. ACCsup, anterior cingulate cortex, supracallosal; MCC, middle cingulate & paracingulate gyri.

For FCs from the bilateral caudate ACC, there was a significant main effect of CT. Individuals with moderate-to-severe CT independent of MDD diagnosis showed increased FC between bilateral caudate ACC and left middle frontal gyrus (MFG) (GRF correction with cluster-level at *P* < 0.05 and a voxel-level threshold of *P* < 0.001) (Figure 2). The main effect of diagnosis was significant (Figure 3). Patients with MDD exhibited decreased FC between the left caudal ACC and left MFG, right SFG, right supramarginal gyrus (SMG) and anterior cingulate cortex, supracallosal (ACCsup) (Figure 3A); between the right caudal ACC and right SFG, right SMG and left ACCsup (Figure 3B); between left dorsal ACC and left MFG, left ACCsup, right SFG and right SMG (Figure 3C); between right dorsal ACC and left MFG, right SFG and right SMG (Figure 3D); between left rostral ACC and left middle cingulate & paracingulate gyri (MCC) and left superior temporal gyrus (STG) (Figure 3E); between right rostral ACC and left MFG (Figure 3F); between left perigenual ACC and left middle temporal gyrus (MTG) and right angular gyrus (ANG) (Figure 3G); between left subgenual ACC and left MTG (Figure 3H). No other significant main effect or interactions of diagnosis-by-CT on FC could be identified (Table 3).

**TABLE 3:** Diagnosis and childhood trauma effects on functional connectivity of anterior cingulate cortex (ACC) subregions.

Seeds	Regions	BA	Cluster size	Peak MNI coordinates			Peak T-value
				**X**	**Y**	**Z**	
**CT main effect**							
**With moderate-to-severe CT > with no or low CT**							
L-caudal ACC	L-MFG	10	190	−42	57	9	5.3987
R-caudal ACC	L-MFG	10	81	−42	57	12	3.744
**Diagnosis main effect**							
**MDD HC**							
L-caudal ACC	L-MFG	10	245	−33	51	33	−5.6905
	R-SFG	10	287	30	48	12	−5.0098
	R-SMG	40	137	63	−36	30	−4.6357
	L-ACCsup	24	103	0	27	18	−4.3193
R-caudal ACC	R-SFG	10	200	30	57	15	−4.7783
	R-SMG	40	194	63	−36	33	−4.4833
	L-ACCsup	24	110	−3	24	18	−4.4963
L-dorsal ACC	L-MFG	10	126	−33	51	33	−6.331
	L-ACCsup	24	146	0	9	33	−4.7657
	R-SFG	10	108	33	45	21	−4.3784
	R-SMG	40	132	63	−33	27	−3.9664
R-dorsal ACC	L-MFG	10	79	−33	51	33	−5.5866
	R-SFG	10	91	30	48	21	−4.3912
	R-SMG	40	88	63	−33	36	−3.8902
L-rostral ACC	L-MCC	24	250	3	−9	39	−4.3242
	L-STG	22	116	−60	−39	15	−4.1172
R-rostral ACC	L-MFG	10	105	−33	51	33	−4.706
L-perigenual	R-ANG	39	149	57	−63	24	−5.5247
	L-MTG	21	109	−63	−57	21	−4.3724
L-subgenual	L-MTG	39	124	−48	−39	18	−4.2393

MDD, major depressive disorder; HC, healthy control; L, left; R, right; ACC, anterior cingulate cortex; SFG, superior frontal gyrus; MFG, middle frontal gyrus; SMG, supramarginal gyrus; STG, superior temporal gyrus; MTG, middle temporal gyrus; ANG, angular gyrus; ACCsup, anterior cingulate cortex supracallosal; MCC, middle cingulate & paracingulate gyri.

### Correlation and mediation analysis in the MDD group

Given that the main effect of CT on HAMD-17 total score and HAMD-cognitive disturbance factor was detected, partial correlation and mediation analyses were performed to investigate the correlation between the altered FCs and the main effect of CT on the HAMD-17 total score and CTQ total score and subscales scores in MDD groups with age, gender, years of education, duration of illness, and mean FD included as covariates.

**FIGURE 4:**
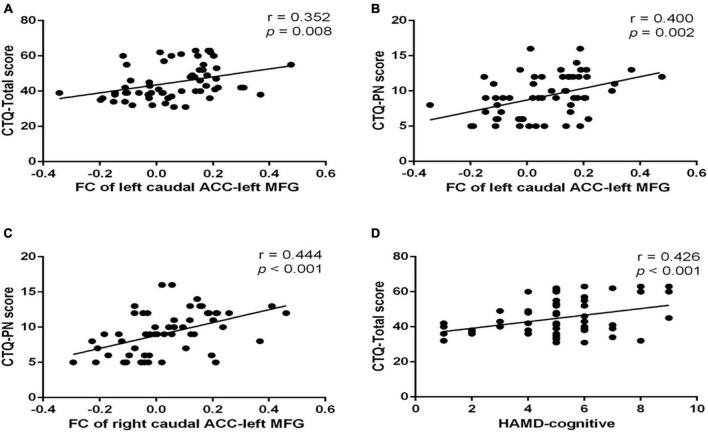
Scatter plots of partial correlation between significant FC and the target scales in MDD group. **(A)** Positive partial correlation between FC of left caudal ACC to left MFG and CTQ total score. **(B)** Positive partial correlation between FC of left caudal ACC to left MFG and CTQ-Physical neglect score. **(C)** Positive partial correlation between FC of right caudal ACC to left MFG and CTQ-Physical neglect score. **(D)** Positive partial correlation between HAMD-cognitive and CTQ-Total score. All correlations showed in this figure were constructed after controlling gender, age, years of education, duration of illness, and mean frame displacement value. MDD, major depressive disorder; FC, functional connectivity; CTQ, Childhood Trauma Questionnaire; CTQ-EN, emotional neglect subscale of childhood trauma questionnaire; CTQ-PN, physical neglect subscale of childhood trauma questionnaire; ACC, anterior cingulate cortex; MFG, middle frontal gyrus.

**FIGURE 5:**
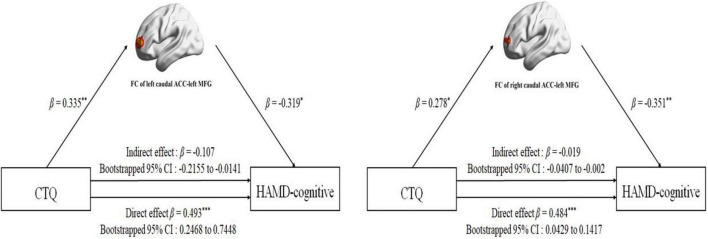
Mediation analysis for effect of the FC between bilateral caudal ACC and left MFG on the relationship between childhood traumatic (CTQ total score) and HAMD-cognitive disturbance factor (HAMD-cognitive score) in MDD group. For the path coefficient: *p < 0.05; **p < 0.01; ***p < 0.001. CTQ, Childhood Trauma Questionnaire; HAMD-cognitive, Hamilton Depression Scale-cognitive disturbance factor; FC, functional connectivity; ACC, anterior cingulate cortex; MFG, middle frontal gyrus; β, standardized regression coefficient; Cl, confidence interval.

The FC between the left caudal ACC and the left MFG was positively correlated with CTQ-physical neglect score (r = 0.400, *p* = 0.002) (Figure 4A) and CTQ total score (r = 0.352, *p* = 0.008) (Figure 4B). The FC between the right caudal ACC and the left MFG was also positively correlated with CTQ-physical neglect score (r = 0.444, *p* < 0.001) (Figure 4C). The HAMD-cognitive score was positively correlated with CTQ total score (r = 0.426, *p* < 0.001) (Figure 4D). In the mediation analysis, the FC of left caudal ACC to left MFG partially mediates the relationship between CTQ total score and HAMD-cognitive disturbance factor in MDD patients (indirect effect: β = −0.107, bootstrapped 95% CI = −0.2155 to −0.0141; direct effect: β = 0.493, bootstrapped 95% CI = 0.2468–0.7448) with covariates of gender, age, education level, mean FD, and duration of illness; the FC of right caudal ACC to left MFG partially mediates the relationship between CTQ total score and HAMD-cognitive disturbance factor in MDD patients (indirect effect: β = −0.019, bootstrapped 95% CI = −0.0407 to −0.002; direct effect: β = 0.484, bootstrapped 95% CI = 0.0429–0.1417) with covariates of gender, age, education level, mean FD, and duration of illness (Figure 5). The complete list of partial correlation and mediation analyses is presented in the Supplementary material.

## Discussion

This present study investigated the effects of CT severity and MDD on seed-to-voxel FC patterns of ACC subregions and the association among childhood trauma, altered FCs, and depressive symptoms. Our preliminary findings showed that there was a significant main effect of diagnosis and CT severity level, but no interaction effect between MDD and CT severity level on the FC of ACC subregions. Specifically, regardless of the diagnosis of MDD, individuals with moderate-to-severe CT exhibited increased FC between bilateral caudal ACC and left MFG relative to individuals with no or low CT. FC of the caudal ACC to MFG mediated the association between CTQ and the cognitive disturbance symptoms of MDD. In terms of the main effect of MDD diagnosis compared to the HC, the caudal ACC and dorsal ACC exhibited a reduction in FC within the regions of the cognitive control network (CCN), including the SFG, MFG, and SMG in MDD patients. In addition, the subgenual and perigenual ACC exhibited attenuated FC the ANG and MTG, which are the key regions of the DMN.

An important finding was that individuals with moderate to severe CT showed a significantly increased FC between the bilateral caudal ACC and MFG compared to the individuals with no or low CT. Caudal ACC plays a crucial role in conflict monitoring (51–53). MFG, which is part of the dorsolateral prefrontal cortex, is mainly involved in higher-order cognitive functions such as working memory and executive control (54). An influential theoretical framework proposed that the ACC detects potential conflicts and subsequently relays them to the DLPFC to implement execution control processes (55, 56). Stress and emotion-processing brain regions may be permanently altered as a result of traumatic experiences in childhood, resulting in greater sensitivity to stressful situations in adulthood (57). Higher FC between the caudal ACC and DLPFC may represent high demands of executive control function (decreased neural efficiency/greater effort) in individuals with moderate and severe CT. This finding indicated that individuals exposed to a higher level of CT often require a greater level of behavioral flexibility to adapt to changing environments and come up with alternate solutions to problems. The findings of this study are in agreement with those of several previous studies. Mueller et al. (58) reported that adolescents with early-life stress exhibited increased activity in brain regions related to executive control. Moreover, we found there is no alteration of FC in other ACC subdivisions in individuals with CT, suggesting that CCN might be a potential biomarker for CT.

In contrast to the increased FC of the left caudal ACC to the left MFG, our results demonstrated decreased FC of the left caudal ACC to the left MFG. Having minimal executive control is associated with emotional regulation difficulties, ruminating, and a reduction in social skills, which are all predictive of psychopathology (59). Thus, the caudal ACC and MFG connectivity disruptions may impair executive control function and lead to rumination in MDD patients. Combining the above findings, the difference in FC patterns between the caudal ACC and MFG may hint at maltreatment-related changes in executive control networks and not just an epiphenomenon of concurrent MDD (60). Moreover, we also found that the FC between the caudal ACC and DLPFC partially mediated the CTQ total score and HAMD-cognitive disturbance factor. This mediation analysis indicated that the impairment of executive control function caused by childhood trauma may embed latent vulnerability to MDD.

In our current study, compared with HCs, MDD patients showed a reduction in FC between the bilateral dorsal ACC and left MFG, right SFG and right SMG. Dorsal ACC is responsible for coordinating and integrating information to guide behavior. The SFG and MFG are located within the DLPFC that is involved in planning complex cognitive behavior, making decisions, and regulating emotions (61). The SMG is a part of the inferior parietal lobule (IPL). The CCN, which is mainly responsible for aspects of cognitive processing such as working memory, decision-making, and attentional allocation, is composed of the dorsal ACC, DLPFC, and IPL (62–65). The reduction in FC between the dorsal ACC and left MFG, right SFG and right SMG indicates a functional disruption within the CCN in MDD patients, which may contribute to MDD patients’ difficulty in ignoring negative valence and stimuli from entering and remaining in the working memory, leading to rumination, which is a core feature of MDD (31). These findings are consistent with prior studies (16, 66, 67). Furthermore, previous studies have observed that the increased metabolic CCN is responsible for the remission of depression (66, 68, 69). This suggested that the important role of dACC activity in depression physiopathology.

This present study demonstrated that both attenuated FC between the perigenual/subgenual ACC and MTG and reduction in FC between the perigenunal ACC and ANG were found in MDD patients relative to HCs. The perigenual and subgenual ACC belong to the affective division of the ACC, which is an essential component of the affective network (AN) (70). The AN is presumed to be involved in emotional processing including fear, vigilance, and autonomic and visceral regulation (71, 72). The MTG and ANG are key hubs of the DMN which is responsible for emotion regulation, future planning, and self-observation (73). In MDD, the decreased FC between the subgenual/perigenual ACC and MTG can disrupt the communication of the AN to the DMN and result in emotional regulation deficits. Several previous studies have supported these observations. One researcher found that MDD patients showed decreased subgenual ACC FC with the MTG; angular gyrus and posterior cingulate cortex (PCC) were related to higher depressive symptoms (74). Peng et al. (37) found a reduction in FC between the perigenual ACC and the DMN.

Currently, this is the first study examining the effect of MDD and CT on the FC of ACC subregions in MDD patients and HCs (with moderate-to-severe and with no or low CT). We used this study design to investigate separately as well as jointly the effects of MDD and CT on the FC of ACC subregions. Moreover, all MDD patients were medication-naive, removing the possibility of medication effects confounding the results. However, this study has several limitations. The severity of trauma was primarily determined by self-reported CTQ score, which may be influenced by recall bias; however, recent studies have demonstrated that subjective self-reported maltreatment is a reliable predictor of psychopathology (75). Since the results presented in the current study are based on the ROI-based FC, we may overlook potential findings. Other ROI-free FC analysis methods [e.g., functional connectivity density (FCD) (76)] should be considered in future studies. Neuroticism is an important risk factor for the development of MDD. A recent meta-analysis demonstrated that neuroticism was positively correlated with the activity of the subgenual ACC (77). Future studies should assess CT and the level of neuroticism in MDD patients, and then clarify more clearly the effect of CT on ACC functional activities in MDD without the influence of neuroticism. These findings should be interpreted with caution due to the relatively small samples of subjects in the MDD patients with low or no CT and HCs with moderate-to-severe CT groups; thus, it is recommended that more patients be included in future studies. Moreover, a high level of anxiety symptoms in MDD patients might have possibly affected these results in the current study; however, there was no significant correlation between the HAMA scores and the ACC subregions FC. Patients with depression without anxiety symptoms should be recruited to validate the findings of the current study in the future. Finally, this study is cross-sectional, which makes causality determination difficult; a longitudinal design would be particularly helpful in future research to clarify the way CT affects depression onset.

## Conclusion

In summary, this study investigated the FC of ACC subdivisions in first-episode, drug-naive MDD patients with moderate-to-severe and with no or low CT. Compared to the HCs, MDD patients showed decreased FC of the caudal ACC/dorsal ACC and the regions of the CCN (SFG, MFG, and SMG), and reduction in FC between the subgenual/perigenual ACC and the key regions of the DMN (ANG and MTG) indicated that depression might be caused by abnormal functional interactions between the brain areas in both the DMN and CCN. Individuals with moderate-to-severe CT demonstrated an increased FC of caudal ACC-MFG, indicating that the disrupted functional integration of caudal ACC may be characteristic of CT. Furthermore, enhanced FC of caudal ACC-MFG mediated the association between CT and depression, suggesting that the aberrant functional integration between caudal ACC and MFG is pivotal in understanding the link between depression and CT. In light of these findings, it may be possible to develop a better understanding of the roles of specific ACC subregions in MDD pathophysiology and the neurophysiological basis of the association between CT and MDD.

## Data availability statement

The original contributions presented in this study are included in the article/Supplementary material, further inquiries can be directed to the corresponding authors.

## Ethics statement

The studies involving human participants were reviewed and approved by the Ethics Committee of the Renmin Hospital of Wuhan University (WDRY2020-K236). The patients/participants provided their written informed consent to participate in this study.

## Author contributions

GW and LX designed the current study. BR drafted the manuscript. BR, GG, LS, MZ, HZ, and JH performed the experiments. BR and HW analyzed the data. BR, GG, LX, and GW revised the manuscript. All authors read and approved the final manuscript.

## Footnotes

1
http://www.fil.ion.ucl.ac.uk/spm
