# Supplementary material for: Preliminary findings on the effect of childhood trauma on the functional connectivity of the anterior cingulate cortex subregions in major depressive disorder
Source: Front Psychiatry. 2023 Apr 17;14:1159175. doi: 10.3389/fpsyt.2023.1159175 (PMC10150086; doi:10.3389/fpsyt.2023.1159175)
Supplement: Supplementary file 1 [file Data_Sheet_1.docx]

Supplementary Material

**Preliminary effects of childhood trauma on functional connectivity of the anterior cingulate cortex subregions major depressive disorder**

Bei Rong^*^, Guoqing Gao, Limin Sun, Mingzhe Zhou, Haomian Zhao, Junhua Huang, Ling Xiao, Gaohua Wang

*** Correspondence:**Ling Xiao lingxiaoxiao@whu,edu.cn

Gaohua Wang [wgh6402@whu.edu.cn](mailto:wgh6402@whu.edu.cn)

# Supplementary Figures and Tables

## Supplementary Tables

**Table S1** Correlations between functional connectivity of CT main effect and CTQ, anxiety, and

|  | **Left caudal ACC-left MFG** | |  | | **Right caudal ACC-left MFG** | |
| --- | --- | --- | --- | --- | --- | --- |
|  | **r** | ***p*** | |  | **r** | ***p*** |
| **CTQ-EA** | 0.118 | 0.389 | |  | 0.087 | 0.526 |
| **CTQ-PA** | 0.192 | 0.16 | |  | 0.163 | 0.234 |
| **CTQ-SA** | 0.234 | 0.085 | |  | 0.064 | 0.641 |
| **CTQ-EN** | 0.288 | 0.033 | |  | 0.211 | 0.122 |
| **CTQ-PN** | 0.4 | 0.002^a^ | |  | 0.444 | < 0.001^a^ |
| **CTQ** | 0.352 | 0.008^a^ | |  | 0.289 | 0.032 |
| **HAMD-Anxiety** | -0.055 | 0.688 | |  | -0.297 | 0.028 |
| **HAMD-Weight** | 0.179 | 0.192 | |  | 0.097 | 0.48 |
| **HAMD-Cognitive** | -0.134 | 0.331 | |  | -0.202 | 0.138 |
| **HAMD-Retardation** | 0.002 | 0.99 | |  | -0.065 | 0.636 |
| **HAMD-Sleep** | 0.212 | 0.119 | |  | 0.069 | 0.616 |
| **HAMD-17** | 0.057 | 0.682 | |  | -0.166 | 0.226 |
| **HAMA** | -0.048 | 0.73 | |  | -0.289 | 0.091 |

depression level and subscale scores in MDD

^a^, significant after multiple comparison correction of FDR *P* < 0.05.

^a^ Left caudal ACC-left MFG FC was significantly correlated with both CTQ_PN (*r* = 0.4, *p* = 0.002) and CTQ (*r* = 0. 352, *p* = 0.008) scores in MDD; however, right caudal ACC-left MFG FC was significantly correlated with CTQ_PN (r = -0.4, p < 0.001).

**Abbreviations:** MDD, major depressive disorder; HAMD-17, 17-items Hamilton Depression Scale; HAMA, Hamilton Anxiety Scale; CTQ-EA, Childhood Trauma Questionnaire-Emotional Abuse; CTQ-PA, Childhood Trauma Questionnaire-Physical Abuse; CTQ-SA, Childhood Trauma Questionnaire-Sexual Abuse; CTQ-EN, Childhood Trauma Questionnaire-Emotional Neglect; CTQ-PN, Childhood Trauma Questionnaire-Physical Neglect; CTQ, Childhood Trauma Questionnaire total score; HAMD-17, 17-items Hamilton Depression Scale; HAMD-Anxiety, Hamilton Depression Scale-Anxiety/somatization factor; HAMD-weight, Hamilton Depression Scale-Loss of weight factor; HAMD-cognitive, Hamilton Depression Scale-cognitive disturbance factor; HAMD-Retardation, Hamilton Depression Scale-Retardation factor; HAMD-Sleep, Hamilton Depression Scale-Sleep disorder factor; HAMA, Hamilton Anxiety Scale; ACC, anterior cingulate cortex; MFG, middle frontal gyrus.

**Table S2** Correlations between CTQ and subscale scores with HAMD-17 and subscale scores in MDD

|  | **CTQ** | |  | **CTQ_EA** | | |  | **CTQ_PA** | |  | | **CTQ_SA** | |  | **CTQ_EN** | |  | **CTQ_PN** | |
| --- | --- | --- | --- | --- | --- | --- | --- | --- | --- | --- | --- | --- | --- | --- | --- | --- | --- | --- | --- |
|  | ***r*** | ***p*** | ***r*** | | | ***p*** | ***r*** | | ***p*** | | ***r*** | | ***p*** | ***r*** | | ***p*** | ***r*** | | ***p*** |
| **HAMD-17** | 0.123 | 0.371 | | | 0.172 | 0.208 | 0.205 | | 0.133 | | 0.099 | | 0.474 | 0.163 | | 0.236 | 0.197 | | 0.15 |
| **HAMD-Anxiety** | -0.133 | 0.335 | | | -0.026 | 0.85 | 0.148 | | 0.281 | | -0.052 | | 0.704 | -0.143 | | 0.298 | -0.096 | | 0.486 |
| **HAMD-Weight** | 0.073 | 0.597 | | | 0.037 | 0.789 | 0.047 | | 0.731 | | 0.031 | | 0.82 | 0.153 | | 0.266 | 0.099 | | 0.472 |
| **HAMD-Cognitive** | 0.426 | 0.001 ^a^ | | | 0.376 | 0.005 | 0.018 | | 0.895 | | 0.219 | | 0.108 | 0.249 | | 0.067 | 0.394 | | 0.003 |
| **HAMD-Retardation** | 0.055 | 0.69 | | | 0.132 | 0.337 | 0.167 | | 0.223 | | 0.055 | | 0.691 | 0.059 | | 0.67 | 0.109 | | 0.427 |
| **HAMD-Sleep** | -0.153 | 0.266 | | | -0.119 | 0.388 | 0.175 | | 0.201 | | -0.018 | | 0.896 | 0.107 | | 0.436 | -0.028 | | 0.837 |

**Abbreviations:** MDD, major depressive disorder; HAMD-17, 17-items Hamilton Depression Scale; HAMA, Hamilton Anxiety Scale; CTQ-EA, Childhood Trauma Questionnaire-Emotional Abuse; CTQ-PA, Childhood Trauma Questionnaire-Physical Abuse; CTQ-SA, Childhood Trauma Questionnaire-Sexual Abuse; CTQ-EN, Childhood Trauma Questionnaire-Emotional Neglect; CTQ-PN, Childhood Trauma Questionnaire-Physical Neglect; CTQ, Childhood Trauma Questionnaire total score; HAMD-17, 17-items Hamilton Depression Scale; HAMD-Anxiety, Hamilton Depression Scale-Anxiety/somatization factor; HAMD-weight, Hamilton Depression Scale-Loss of weight factor; HAMD-cognitive, Hamilton Depression Scale-cognitive disturbance factor; HAMD-Retardation, Hamilton Depression Scale-Retardation factor; HAMD-Sleep, Hamilton Depression Scale-Sleep disorder factor.

**Table S3** Functional connectivity of CT main effect mediate the association between childhood trauma and depressive symptoms in MDD group

| **X** | **Y** | **FCs** | **Indirect effect** | **Bootstrap**  **95% CI of a*b** | | **Boot SE** |
| --- | --- | --- | --- | --- | --- | --- |
| CTQ | HAMD-Cognitive | Left caudal ACC-left MFG | -0.107 | -0.216 | -0.014 | 0.052 |
| CTQ | HAMD-Cognitive | Right caudal ACC-left MFG | -0.019 | -0.041 | -0.002 | 0.01 |

**Abbreviations:** MDD, major depressive disorder; CTQ, Childhood Trauma Questionnaire total score; HAMD-cognitive, Hamilton Depression Scale-cognitive disturbance factor; ACC, anterior cingulate cortex; MFG, middle frontal gyrus.
